# SOCIAL ISOLATION AMONG THE RECIPIENTS OF THE MINIMUM LIVELIHOOD GUARANTEE PROGRAM IN CHINA

**DOI:** 10.1093/geroni/igz038.1230

**Published:** 2019-11-08

**Authors:** Yalu Zhang, Ada Mui

**Affiliations:** Columbia University, New York City, New York, United States

## Abstract

The eligibility for the minimum livelihood guarantee program in China is generally based on means testing, including a complicated income test and a household registration test. Even though the fiscal budget for this program steadily increased in the last two decades, the number of beneficiaries has decreased in both rural and urban areas since 2011. The recipients were stigmatized because their names are reported in the public domain and are known to their neighbors. Some beneficiaries tended to reduce social participation for fear of losing the eligibilities due to the stricter criteria or the stigma associated with receiving benefits. This paper examined the adverse impact of this welfare program on social isolation among Chinese middle-aged and older adults (n=8,447) using the panel data from the China Health and Retirement Longitudinal Study and the propensity score weighted difference-in-differences method (PSM-DID). The results present disparities between urban and rural older adults—The older program recipients in urban areas significantly reduced social participation by 20.53%, while the adverse impact was not statistically significant among rural recipients. Compared to older adults (aged 60 and above), middle-aged adults (aged between 45 and 60) were not socially isolated due to program enrollment in both rural and urban areas at the 95% confidence level. This paper for the first time addresses the causal relationship between program enrollment and the adverse impact of social isolation. It also alerts policymakers and local program administrators to protect older program recipients from stigma, sense of shame, and reduced social connection.

